# Asynchronous Semantic Background Subtraction

**DOI:** 10.3390/jimaging6060050

**Published:** 2020-06-18

**Authors:** Anthony Cioppa, Marc Braham, Marc Van Droogenbroeck

**Affiliations:** Montefiore Institute, University of Liège, Quartier Polytech 1, Allée de la Découverte 10, 4000 Liège, Belgium; m.braham@uliege.be (M.B.); m.vandroogenbroeck@uliege.be (M.V.D.)

**Keywords:** background subtraction, motion detection, scene labeling, semantic segmentation, video processing

## Abstract

The method of Semantic Background Subtraction (SBS), which combines semantic segmentation and background subtraction, has recently emerged for the task of segmenting moving objects in video sequences. While SBS has been shown to improve background subtraction, a major difficulty is that it combines two streams generated at different frame rates. This results in SBS operating at the slowest frame rate of the two streams, usually being the one of the semantic segmentation algorithm. We present a method, referred to as “Asynchronous Semantic Background Subtraction” (ASBS), able to combine a semantic segmentation algorithm with any background subtraction algorithm asynchronously. It achieves performances close to that of SBS while operating at the fastest possible frame rate, being the one of the background subtraction algorithm. Our method consists in analyzing the temporal evolution of pixel features to possibly replicate the decisions previously enforced by semantics when no semantic information is computed. We showcase ASBS with several background subtraction algorithms and also add a feedback mechanism that feeds the background model of the background subtraction algorithm to upgrade its updating strategy and, consequently, enhance the decision. Experiments show that we systematically improve the performance, even when the semantic stream has a much slower frame rate than the frame rate of the background subtraction algorithm. In addition, we establish that, with the help of ASBS, a real-time background subtraction algorithm, such as ViBe, stays real time and competes with some of the best non-real-time unsupervised background subtraction algorithms such as SuBSENSE.

## 1. Introduction

The goal of background subtraction (shortened to BGS in the following) algorithms is to automatically segment moving objects in video sequences using a background model fed with features, hand-designed or learned by a machine learning algorithm, generally computed for each video frame. Then, the features of the current frame are compared to the features of the background model to classify pixels either in the background or in the foreground. While being fast, these techniques remain sensitive to illumination changes, dynamic backgrounds, or shadows that are often segmented as moving objects.

Background subtraction has been an active field of research during the last years [1]. It was promoted by the development of numerous variations of the GMM [2] and KDE [3] algorithms, and the emergence of innovative algorithms such as SOBS [4], ViBe [5], SuBSENSE [6], PAWCS [7], IUTIS-5 [8], and PCA variants [9,10]. Research in this field can count on large datasets annotated with ground-truth data such as the BMC dataset [11], the CDNet 2014 dataset [12], or the LASIESTA dataset [13], which was an incentive to develop supervised algorithms. In Reference [14], Braham and Van Droogenbroeck were the first to propose a background subtraction method using a deep neural network; this work paved the way to other methods, proposed recently [15,16,17,18]. Methods based on deep learning have better segmentation performances, but they rely on the availability of a fair amount of annotated training data; to some extent, they have lost their ability to deal with any camera operating in an unknown environment. Note however that, in their seminal work [14], Braham and Van Droogenbroeck present a variation of the network that is trained on ground-truth data generated by an unsupervised algorithm, thus requiring no annotations at all; this idea was later reused by Babaee et al. [19].

Rather than building novel complicated methods to overcome problems related to challenging operational conditions such as illumination changes, dynamic backgrounds, the presence of ghosts, shadows, camouflage or camera jitter, another possibility consists in leveraging the information provided by a universal semantic segmentation algorithm for improving existing BGS algorithms. Semantic segmentation of images consists in labeling each pixel of an image with the class of its enclosing object or region. It is a well-covered area of research, but it is only recently that it has achieved the level of performance needed for real applications thanks to the availability of large annotated datasets such as ADE20K [20], VOC2012 [21], Cityscapes [22] or COCO [23], and novel deep neural networks [24,25,26]. In the following, we use the term *semantics* to denote the output of any of these semantic segmentation networks.

The performances achieved by these deep networks for the task of semantic segmentation have motivated their use for various computer vision tasks such as optical flow computation [27], or motion segmentation [28,29]. The underlying idea is to segment objects and characterize their motion using, in our case, background subtraction in video sequences [30]. It is important to note that semantic segmentation algorithms are trained with annotated datasets that contain varied types of objects, most of which do not appear in videos such as those of the CDNet 2014 dataset. In other words, semantic segmentation algorithms are not tailored for the task of motion detection. While this is a suitable feature to deal with arbitrary unknown scenes, it requires to validate if a network works well on the typical images encountered in background subtraction.

Recently, Braham et al. [30] presented the semantic background subtraction method (named SBS hereafter), that leverages semantics for improving background subtraction algorithms. This method, which combines semantics and the output of a background subtraction algorithm, reduces the mean error rate up to 20% for the 5 best unsupervised algorithms on CDNet 2014 [12]. Unfortunately, in practice, it is often much slower to compute semantics than it is to perform background subtraction. Consequently, to avoid reducing the frame rate of the images processed by background subtraction, semantics needs to be computed on a dedicated hardware (such as a modern GPU) and fed asynchronously, that is with missing semantic frames.

### Problem Statement

**Figure 1:**
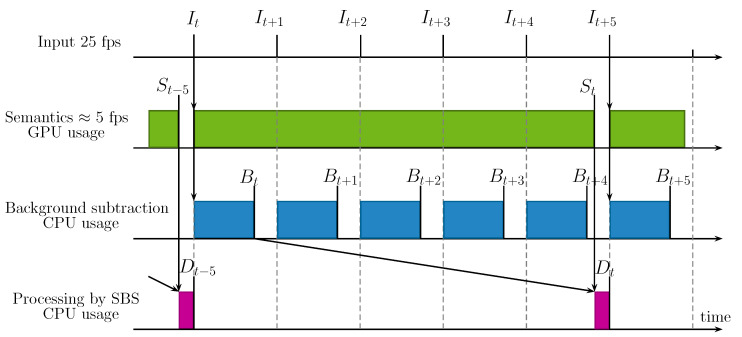
Timing diagram of a naive real-time implementation of the semantic background subtraction (SBS) method when the frame rate of semantics is too slow to handle all the frames in real time. From top to bottom, the time lines represent: the input frames $I_{t}$, the computation of semantics $S_{t}$ by the semantic segmentation algorithm (on GPU), the computation of intermediate segmentation masks $B_{t}$ by the background subtraction (BGS) algorithm (on CPU), and the computation of output segmentation masks $D_{t}$ by the SBS method (on CPU). Vertical lines indicate when an image is available and filled rectangular areas display when a GPU or CPU performs a task. Arrows show the inputs required by the different tasks. This diagram shows that even when the background subtraction algorithm is real time with respect to the input frame rate, it is the computation of semantics that dictates the output frame rate.

To better understand the problem, let us analyze the timing diagram of SBS, as displayed in Figure 1. For this time analysis, we assume that a GPU is used for semantic segmentation, and a CPU is used for both the BGS algorithm and the SBS method. When the GPU is available, it starts analyzing the input frame, otherwise it skips it. In the scenario of a BGS algorithm being faster than the semantic segmentation network, which is the scenario that we examine in this paper, the BGS algorithm starts as soon as the previous processing is over. The CPU then waits until semantics has been computed and a semantic frame $S_{t}$ is available. The timeline analysis of SBS shows that—(1) with respect to the input frame, the output frame is delayed by the time to compute semantics and to process the segmentation map (this delay is unavoidable and constant), and (2) the output frame rate is mainly driven by the slowest operation. It results that some output frames would be skipped, although the CPU computes all the intermediate masks by the BGS algorithm. For example, in the case of Figure 1, it is possible to apply the BGS algorithm to $I_{t+2}$, but not to process $B_{t+2}$ with the help of semantics. In other words, the slowest operation dictates its rhythm (expressed in terms of frame rate) to the entire processing chain. Hence, the semantics and the output have equal frame rates. This is not a problem as long as the output frame rate (or equivalently that of semantics) is faster than the input frame rate. However, the semantics frame rate is generally slower than the input frame rate, which means that it is not possible to process the video at its full frame rate, or in order words, that the processing of SBS is not real time.

To increase the output frame rate to its nominal value, we need to either accelerate the production of semantics, which induces the choice of a faster but less accurate semantic network, or to interpolate the missing semantics. Our analysis on semantic networks showed that faster networks are not exploitable because of their lack of precision. Also, semantic segmentation networks should be preferred to instance segmentation networks. For example, we had to discard MaskRCNN [26] and prefer the PSPNet network [25], as shown in Table 1.

An alternative option is to interpolate missing semantics. Naive ideas would be to skip the SBS processing step in the absence of semantics or to repeat the last pixelwise semantic information when it is missing. Both ideas proved unsuccessful, as shown in our experiments (see Section 4). A better idea is to avoid any mechanism that would substitute itself to the difficult calculation of semantics and, instead, replicate the decisions enforced previously with the help of semantics to compensate for the lack of semantics later on. The underlying question is whether or not we should trust and repeat decisions taken by SBS [30]. This idea has already been applied in one of our recent work, called Real-time Semantic Background Subtraction [31] (noted RT-SBS) with ViBe, a real-time BGS algorithm, and forms the basis of our new method, ASBS. This paper presents our method in a complementary way to the original paper, with further experiments and generalizes it to all background subtraction algorithms, including non-real-time ones.

The paper is organized as follows. Section 2 describes the semantic background subtraction (SBS) method that underpins our developments. In Section 3, we first discuss the classification problem of background subtraction and take into account the specificities of semantics. Then, we describe our new method. Experimental results are provided in Section 4, and compared with those of the original semantic background subtraction method when semantics is missing for some frames. Finally, we conclude in Section 5.

**Contributions.** We summarize our contributions as follows—(i) We propose a novel method, called ASBS, for the task of background subtraction. (ii) We alleviate the problem of the slow computation of semantics by substituting it for some frames with the help of a change detection algorithm. This makes our method usable in real time. (iii) We show that at a semantic frame rate corresponding to real-time computations, we achieve results close to that of SBS, meaning that our substitute for semantics is adequate. (iv) We show that our method ASBS with a real-time BGS algorithm such as ViBe and a simple feedback mechanism achieves performances close to the ones of non real-time state-of-the-art BGS algorithms such as SuBSENSE, while satisfying the real-time constraint.

## 2. Description of the Semantic Background Subtraction Method

Semantic background subtraction (SBS) [30,32] is a method based on semantics provided by deep segmentation networks that enriches the pixel-wise decisions of a background subtraction algorithm. In this section, we detail how SBS uses semantics to improve the classification of a BGS algorithm. This description is necessary as SBS underpins our strategy to improve background subtraction in the absence of semantics for some frames.

SBS combines three results at each pixel (x,y): the original classification result between background (BG) and foreground (FG) at time *t*, as produced by a chosen BGS algorithm, denoted by $B_{t}\in \left\{\mathrm{BG},\mathrm{FG}\right\}$, and two booleans based on the semantic signals $S_{t}^{\mathrm{BG}}\in \left[0,1\right]$ and $S_{t}^{\mathrm{FG}}\in \left[-1,1\right]$, derived from a semantic probability estimate defined hereinafter. These results are then combined to output the final result $D_{t}\in \left\{\mathrm{BG},\mathrm{FG}\right\}$, as detailed in Table 2.

The two semantic signals ($S_{t}^{\mathrm{BG}}$ and $S_{t}^{\mathrm{FG}}$) are derived from a semantic probability estimate at each pixel location, denoted by $p_{S,t}\left(x,y\right)$. This value is an estimate of the probability that pixel (x,y) belongs to one of the objects contained in a set of potentially moving objects (person, car, etc.) and depends on the segmentation network itself. The authors of Reference [30] use the PSPNet [25] semantic segmentation network and compute $p_{S,t}\left(x,y\right)$ by applying a softmax function on the vector of output scores for this pixel and add up the obtained values for the subset of classes of interest (see Section 4.1 for more implementation details).

The first semantic signal, $S_{t}^{\mathrm{BG}}\left(x,y\right)$, is the semantic probability estimate itself—$S_{t}^{\mathrm{BG}}\left(x,y\right)=p_{S,t}\left(x,y\right)$. It has a low value when the probability is close to zero, meaning that there is no object of interest for that pixel. According to rule1, if this signal is lower than a threshold $\tau_{\mathrm{BG}}$, the pixel is labeled as background:

(1) $$\mathrm{rule}\;1:\mathrm{if}\;\;S_{t}^{\mathrm{BG}}\left(x,y\right)\leq \tau_{\mathrm{BG}},\;\;\mathrm{then}\;\;D_{t}\left(x,y\right)\leftarrow \mathrm{BG}\;.$$

A convenient interpretation of rule1 is that when it is activated (that is, when the condition is true), the decision of the BGS algorithm is shadowed. Consequently, the amount of false positives (pixels wrongly classified in the foreground), typically generated by illumination changes, dynamic backgrounds or the presence of ghosts, is reduced since the semantic segmentation is unaffected by these well-known BGS problems.

The second semantic signal, $S_{t}^{\mathrm{FG}}\left(x,y\right)$, aims at improving the detection of foreground objects by detecting a local increase of the semantic probability estimate compared to a semantic background model, denoted by $M_{t}$. The signal $S_{t}^{\mathrm{FG}}$ is calculated as the difference between the current semantic probability estimate and the value stored in the semantic background model:

(2) $$S_{t}^{\mathrm{FG}}\left(x,y\right)=p_{S,t}\left(x,y\right)-M_{t}\left(x,y\right)\;,$$

where the semantic background model $M_{t}$ is initialized via:

(3) $$M_{0}\left(x,y\right)\leftarrow p_{S,0}\left(x,y\right)\;,$$

and is possibly updated for each pixel only if the pixel is classified as belonging to the background:

(4) $$\begin{array}{cc} \mathrm{if}\;\;D_{t}\left(x,y\right)=\mathrm{BG}, & \;\;\mathrm{then}{}_{\alpha }\;\;M_{t+1}\left(x,y\right)\leftarrow p_{S,t}\left(x,y\right)\;, \end{array}$$

with the expression “$\mathrm{if}\;\;A\;\;{\mathrm{then}}_{\alpha }\;\;B$” meaning that action *B* is applied with a probability α if condition *A* is true. The goal for $M_{t}\left(x,y\right)$ is to store the semantic probability estimate of the background in that pixel. When the value of $S_{t}^{\mathrm{FG}}\left(x,y\right)$ is large, a jump in the semantic probability estimate for pixel (x,y) is observed, and we activate rule2 as defined by:

(5) $$\mathrm{rule}\;2:\mathrm{if}\;\;S_{t}^{\mathrm{FG}}\left(x,y\right)\geq \tau_{\mathrm{FG}},\;\;\mathrm{then}\;\;D_{t}\left(x,y\right)\leftarrow \mathrm{FG}\;,$$

where $\tau_{\mathrm{FG}}$ is a second positive threshold.

Again, when the condition of rule2 is fulfilled, the result of the BGS algorithm is shadowed. This second rule aims at reducing the number of missing foreground detections, for example when a foreground object and the background appear to have similar colors (this is known as the color camouflage effect). Note that, with a proper choice of threshold values $\tau_{\mathrm{BG}}<\tau_{\mathrm{FG}}$, both rules are fully compatible meaning that they are never activated simultaneously. This relates to the “don’t-care” situations described in Table 2.

The decision table of Table 2 also shows that, when none of the two rules are activated, we use the result of the companion BGS algorithm as a fallback decision:

(6) $$\mathrm{fallback}:D_{t}\left(x,y\right)\leftarrow B_{t}\left(x,y\right).$$

## 3. Asynchronous Semantic Background Subtraction

To combine the output of any background subtraction to semantics according to SBS in real time, it is necessary to calculate semantics at least at the same frame rate as the input video or BGS stream, which is currently not achievable with high performances on any kind of videos, even on a GPU. Instead of lowering the frame rate or reducing the image size, an alternative possibility consists to interpolate missing semantics. Naive ideas, such as skipping the combination step of SBS in the absence of semantics or repeating the last pixelwise semantic information when it is missing, have proved unsuccessful, as shown in our experiments (see Section 4). Hence, it is better to find a substitute for missing semantics. Obviously, it is unrealistic to find a substitute that would be as powerful as full semantics while being faster to calculate. Instead, we propose to replicate the decisions enforced previously with the help of semantics to compensate for the lack of semantics later on. The underlying question is whether or not we should trust and repeat decisions taken by SBS [30]. This idea is the basis of our new method.

The cornerstone for coping with missing semantics is the fact that the true class (foreground or background) of a pixel generally remains unchanged between consecutive video frames, as long as the object in that pixel remains static. It is therefore reasonable to assume that if a correct decision is enforced with the help of semantics for a given pixel location and video frame, the same decision should be taken in that pixel location for the subsequent frames (when semantics is not computed) if the features of that pixel appear to be unchanged. Our method, named Asynchronous Semantic Background Subtraction (ASBS), thus consists in interpolating the decisions of SBS by memorizing information about the activation of rules as well as the pixel features, which we chose to be the input color in our case, when semantics is computed (SBS is then applied), and copying the decision of the last memorized rule when semantics is not computed if the color remains similar (which tends to indicate that the object is the same).

To further describe ASBS, let us first focus on a substitute for rule1, denoted ruleA hereafter, that replaces rule1 in the absence of semantics. If rule1 was previously activated in pixel (x,y) while the current color has remained similar, then $D_{t}\left(x,y\right)$ should be set to the background. To enable this mechanism, we have to store, in a rule map denoted by *R*, if rule1 of SBS is activated; this is indicated by R(x,y)←1. Simultaneously, we memorize the color of that pixel in a color map, denoted by *C*. With these components, ruleA becomes:

$$\mathrm{rule}\;A:\;\mathrm{if}\;R\left(x,y\right)=1\;\;\;\mathrm{and}\;\;\;\mathrm{dist}\left(C\left(x,y\right),I_{t}\left(x,y\right)\right)\leq \tau_{A},$$

(7) $$\;\;\mathrm{then}\;\;D_{t}\left(x,y\right)\leftarrow \mathrm{BG}\;,$$

where $\tau_{A}$ is a fixed threshold applied on the Manhattan (or Euclidean) distance between the color C(x,y) stored in the color map and the input color $I_{t}\left(x,y\right)$. Theoretically, it is also possible to refine the color model by adopting a model used by a BGS algorithm in which case the distance function should be chosen accordingly; our choice to favor a simple model instead proved effective.

Likewise, we can replace rule2 by ruleB in the absence of semantics. When rule2 is activated, this decision is stored in the rule map (this is indicated by R(x,y)←2), and the color of the pixel is stored in the color map *C*. RuleB thus becomes:

$$\mathrm{rule}\;B:\;\mathrm{if}\;R(x,y)=2\;\;\;\mathrm{and}\;\;\;\mathrm{dist}\left(C\left(x,y\right),I_{t}\left(x,y\right)\right)\leq \tau_{B},$$

(8) $$\;\;\mathrm{then}\;\;D_{t}\left(x,y\right)\leftarrow \mathrm{FG}\;,$$

where $\tau_{B}$ is a second threshold. Again, when neither ruleA nor ruleB are activated, the BGS decision is used as a fallback decision.

The updates of the rules and color map are detailed in Algorithm 1. It is an add-on for SBS that memorizes decisions and colors based on computed semantics upon activation of a rule. The second component of ASBS, described in Algorithm 2, is the application of ruleA, ruleB, or the fallback decision, when no semantics is available.

**Algorithm 1** Pseudo-code of ASBS for pixels with semantics. The rule and color maps are updated during the application of SBS (note that *R* is initialized with zero values at the program start).**Require:**$I_{t}$ is the input color frame (at time *t*) 1:  **for all**
(x,y) with semantics **do** 2:  $D_{t}\left(x,y\right)\leftarrow apply\;\mathrm{SBS}\;in\;\left(x,y\right)$ 3:  **if**
rule1 was activated **then** 4:    R(x,y)←1 5:    $C\left(x,y\right)\leftarrow I_{t}\left(x,y\right)$ 6:  **else if**
rule2 was activated **then** 7:    R(x,y)←2 8:    $C\left(x,y\right)\leftarrow I_{t}\left(x,y\right)$ 9:  **else** 10:    R(x,y)←0 11:  **end if** 12:  **end for**

**Algorithm 2** Pseudo-code of ASBS for pixels without semantics, ruleA, ruleB or the fallback are applied.**Require:**$I_{t}$ is the input color frame (at time *t*) 1:**  for all**
(x,y) without semantics **do** 2:  **if**
R(x,y)=1
**then** 3:    **if**
$\mathrm{dist}\left(C\left(x,y\right),I_{t}\left(x,y\right)\right)\leq \tau_{A}$
**then** 4:      $D_{t}\left(x,y\right)\leftarrow \mathrm{BG}$ 5:    **end if** 6:  **else if**
R(x,y)=2
**then** 7:    **if**
$\mathrm{dist}\left(C\left(x,y\right),I_{t}\left(x,y\right))\right)\leq \tau_{B}$
**then** 8:      $D_{t}\left(x,y\right)\leftarrow \mathrm{FG}$ 9:    **end if** 10:  **else** 11:    $D_{t}\left(x,y\right)\leftarrow B_{t}\left(x,y\right)$ 12:  **end if** 13:  **end for**

**Figure 2:**
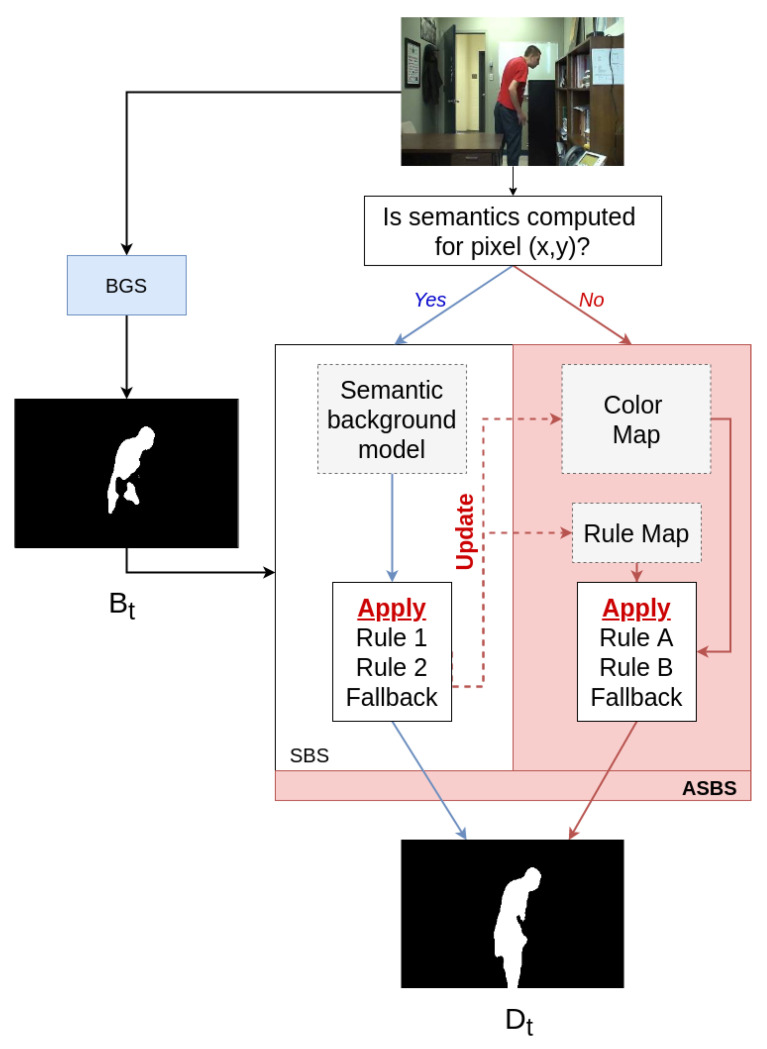
Schematic representation of our method named ASBS, extending SBS [30], capable to combine the two asynchronous streams of semantics and background subtraction masks to improve the performances of BGS algorithms. When semantics is available, Asynchronous Semantic Background Subtraction (ASBS) applies Rule 1, Rule 2, or selects the fallback, and it updates the color and rule maps. Otherwise, ASBS applies Rule A, Rule B, or it selects the fallback.

Note that the two pseudo-codes, which define pixel-wise operations, could be applied within the same video frame if the semantics was only computed inside a specific region-of-interest. In that scenario, we would apply the pseudo-code of Algorithm 2 for pixels without semantics and the pseudo-code of Algorithm 1 for pixels with semantics. It is therefore straightforward to adapt the method from a temporal sub-sampling to a spatial sub-sampling, or to a combination of both. However, a typical setup is that semantics is computed for the whole frame and is skipped for the next few frames at a regular basis. In Section 4, we evaluate ASBS for this temporal sub-sampling since it has a unique implementation, while spatial sub-sampling can involve complex strategies for choosing the regions where to compute the semantics and is application-dependent anyway. Our method, illustrated in Figure 2 for the case of entire missing semantic frames, is applicable in combination with virtually any BGS algorithm.

### Timing Diagrams of ASBS

The ASBS method introduces a small computational overhead (a distance has to be computed for some pixels) and memory increase (a rule map and a color map are memorized). However, these overheads are negligible with respect to the computation of semantics. The practical benefits of ASBS can be visualized on a detailed timing diagram of its components. For a formal discussion, we use the following notations:

- $I_{t}$, $S_{t}$, $B_{t}$, $D_{t}$ respectively denote an arbitrary input, semantics, background segmented by the BGS algorithm, and the background segmented by ASBS, indexed by *t*.
- $\delta_{I}$ represents the time between two consecutive input frames.
- $\Delta_{S}$, $\Delta_{B}$, $\Delta_{D}$ are the times needed to calculate the semantics, the BGS output, and to apply SBS or ASBS, which are supposed to be the same, respectively. These times are reasonably constant.

We assume that semantics is calculated on a GPU, whereas the BGS and the application of the rules are calculated on a single threaded CPU hardware. Also, the frame rate of semantics is supposed to be smaller than that of BGS; that is $\Delta_{S}>\Delta_{B}$.

**Figure 3:**
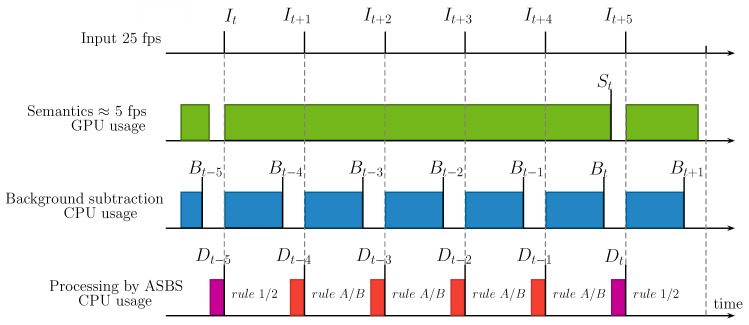
Timing diagram of ASBS in the case of a real-time BGS algorithm ($\Delta_{B}<\delta_{I}$) satisfying the condition $\Delta_{B}+\Delta_{D}<\delta_{I}$. Note that the output stream is delayed by a constant $\Delta_{S}+\Delta_{D}$ time with respect to the input stream.

We now examine two different scenarios. The first scenario is that of a real-time BGS algorithm ($\Delta_{B}<\delta_{I}$) satisfying the condition $\Delta_{B}+\Delta_{D}<\delta_{I}$. This scenario, illustrated in Figure 3, can be obtained with the ViBe [5] BGS algorithm for example; this scenario is further described in Reference [31]. On the timing diagram, it can be seen that the output frame rate is then equal to the input frame rate, all frames being segmented either by SBS (rule 1/2) or ASBS (rule A/B) with a time delay corresponding approximately to $\Delta_{S}$. We present illustrative numbers for this timing diagram in Section 4.4.

In a second scenario, the frame rate of the BGS is too slow to accommodate to real time with ASBS. It means that $\Delta_{B}+\Delta_{D}>\delta_{I}$. In this case, the output frame rate is mainly dictated by $\Delta_{B}$, since $\Delta_{B}>>\Delta_{D}$. The input frame rate can then be viewed as slowed down by the BGS algorithm, in which case the timing diagrams fall back to the same case as a real-time BGS algorithm by artificially changing $\delta_{I}$ to $\tilde{\delta_{I}}$, where $\tilde{\delta_{I}}=\Delta_{B}+\Delta_{D}>\delta_{I}$. It is a scenario that, unfortunately, follows the current trend to produce better BGS algorithms at the price of more complexity and lower processing frame rates. Indeed, according to our experiments and Reference [33], the top unsupervised BGS algorithms ranked on the CDNet web site (see http://changedetection.net) are not real time.

## 4. Experimental Results

In this section, we evaluate the performances of our novel method ASBS and compare them to those of the original BGS algorithm and those of the original SBS method [30]. First, in Section 4.1, we present our evaluation methodology. This comprises the choice of a dataset along with the evaluation metric, and all needed implementation details about ASBS, such as how we compute the semantics, and how we choose the values of the different thresholds. In Section 4.2, we evaluate ASBS when combined with state-of-the-art BGS algorithms. Section 4.3 is devoted to a possible variant of ASBS which includes a feedback mechanism that can be applied to any conservative BGS algorithm. Finally, we discuss the computation time of ASBS in Section 4.4.

### 4.1. Evaluation Methodology

For the quantitative evaluation, we chose the CDNet 2014 dataset [12] which is composed of 53 video sequences taken in various environmental conditions such as bad weather, dynamic backgrounds and night conditions, as well as different video acquisition conditions, such as PTZ and low frame rate cameras. This challenging dataset is largely employed within the background subtraction community and currently serves as the reference dataset to compare state-the-art BGS techniques.

We compare performances on this dataset according to the overall $F_{1}$ score, which is one of the most widely used performance scores for this dataset. For each video, $F_{1}$ is computed by:

(9) $$F_{1}=\frac{2TP}{2TP+FP+FN}\;,$$

where TP (true positives) is the number of foreground pixels correctly classified, FP (false positives) the number of background pixels incorrectly classified, and FN (false negatives) the number of foreground pixels incorrectly classified. The overall $F_{1}$ score on the entire dataset is obtained by first averaging the F1 scores over the videos, then over the categories, according the common practice of CDNet [12]. Note that this averaging introduces inconsistencies between overall scores that can be avoided by using summarization instead, as described in Reference [34], but to allow a fair comparison with the other BGS algorithms, we decided to stick to the original practice of Reference [12] for our experiments.

We compute the semantics as in Reference [30], that is with the semantic segmentation network PSPNet [25] trained on the ADE20K dataset [35] (using the public implementation [36]). The network outputs a vector containing 150 real numbers for each pixel, where each number is associated to a particular object class within a set of 150 mutually exclusive classes. The semantic probability estimate $p_{S,t}\left(x,y\right)$ is computed by applying a softmax function to this vector and summing the values obtained for classes that belong to a subset of classes that are relevant for motion detection. We use the same subset of classes as in Reference [30] (person, car, cushion, box, boot, boat, bus, truck, bottle, van, bag and bicycle), whose elements correspond to moving objects of the CDNet 2014 dataset.

For dealing with missing semantics, since the possibilities to combine spatial and temporal sampling schemes are endless, we have restricted the study to the case of a temporal sub-sampling of one semantic frame per *X* original frames; this sub-sampling factor is referred to as X:1 hereafter. In other scenarios, semantics could be obtained at a variable frame rate or for some variable regions of interest, or even a mix of these sub-sampling schemes.

The four thresholds are chosen as follows. For each BGS algorithm, we optimize the thresholds $\left(\tau_{\mathrm{BG}},\tau_{\mathrm{FG}}\right)$ of SBS with a grid search to maximize its overall $F_{1}$ score. Then, in a second time, we freeze the optimal thresholds $\left(\tau_{\mathrm{BG}}^{*},\tau_{\mathrm{FG}}^{*}\right)$ found by the first grid search and optimize the thresholds $\left(\tau_{A},\tau_{B}\right)$ of ASBS by a second grid search for each pair (BGS algorithm, X:1), to maximize the overall $F_{1}$ score once again. Such methodology allows a fair comparison between SBS and ASBS as the two techniques use the same common parameters $\left(\tau_{\mathrm{BG}}^{*},\tau_{\mathrm{FG}}^{*}\right)$ and ASBS is compared to an optimal SBS method. Note that the α parameter is chosen as in Reference [30].

The segmentation maps of the BGS algorithms are either taken directly from the CDNet 2014 website (when no feedback mechanism is applied) or computed using the public implementations available in Reference [37] for ViBe [5] and Reference [38] for SuBSENSE [6] (when the feedback mechanism of Section 4.3 is applied).

### 4.2. Performances of ASBS

**Figure 4:**
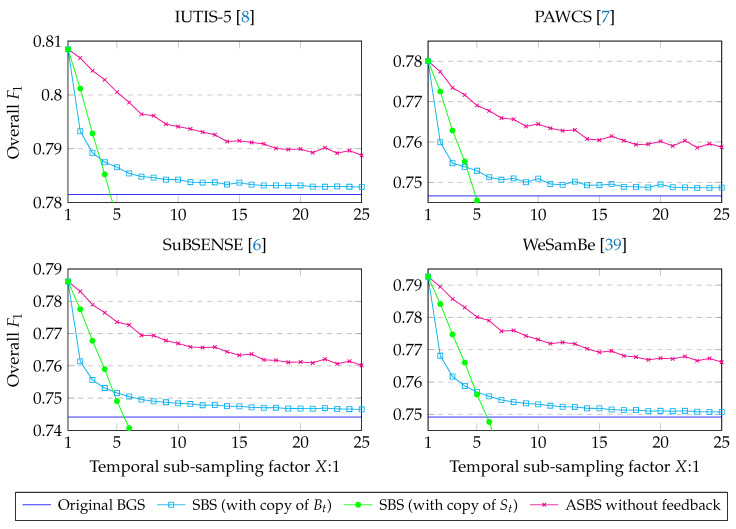
Overall $F_{1}$ scores obtained with SBS and ASBS for four state-of-the-art BGS algorithms and different sub-sampling factors. The performances of ASBS decrease much more slowly than those of SBS with the decrease of the semantic frame rate and, therefore, are much closer to those of the ideal case (SBS with all semantic maps computed, that is SBS 1:1), meaning that ASBS provides better decisions for frames without semantics. On average, ASBS with 1 frame of semantics out of 25 frames (ASBS 25:1) performs as well as SBS, with copy of $B_{t},$ with 1 frame of semantics out of 2 frames (SBS 2:1).

A comparison of the performances obtained with SBS and ASBS for four state-of-the-art BGS algorithms (IUTIS-5 [8], PAWCS [7], SuBSENSE [6], and WebSamBe [39]) and for different sub-sampling factors is provided in Figure 4. For the comparison with SBS, we used two naive heuristics for dealing with missing semantic frame as, otherwise, the evaluation would be done on a subset of the original images as illustrated in Figure 1. The first heuristic simply copies $B_{t}$ in $D_{t}$ for frames with missing semantics. The second heuristic uses the last available semantic frame $S_{t}$ in order to still apply rule1 and rule2 even when no up-to-date semantic frames are available. Let us note that this last naive heuristic corresponds to using ASBS with $\tau_{A}$ and $\tau_{B}$ chosen big enough so that the condition on the color of each pixel is always satisfied.

As can be seen, the performances of ASBS decrease much more slowly than those of SBS with the decrease of the semantic frame rate and, therefore, are much closer to those of the ideal case (SBS with all semantic maps computed, that is SBS 1:1), meaning that ASBS provides better decisions for frames without semantics.

A second observation can be made concerning the heuristic repeating $S_{t}$. The performances become worse than the ones of the original BGS for semantic frame rates lower than 1 out of 5 frames, but they are better than SBS when repeating $B_{t}$ for high semantic frame rates. This observation emphasizes the importance of checking the color feature as done with ASBS instead of blindly repeating the corrections induced by semantics. The performances for lower frame rates are not represented for the sake of figure clarity but still decrease linearly to very low performances. For example, in the case of IUTIS_5, the performance drops to 0.67 at 25:1. In the rest of the paper, when talking about performances on SBS at different frame rates, we only consider the heuristic where we copy $B_{t}$ as it is the one that behaves the best, given our experimental setup. Finally, it can be seen that, on average, ASBS with 1 frame of semantics out of 25 frames (ASBS 25:1) performs as well as SBS, with copy of $B_{t},$ with 1 frame of semantics out of 2 frames (SBS 2:1).

**Figure 5:**
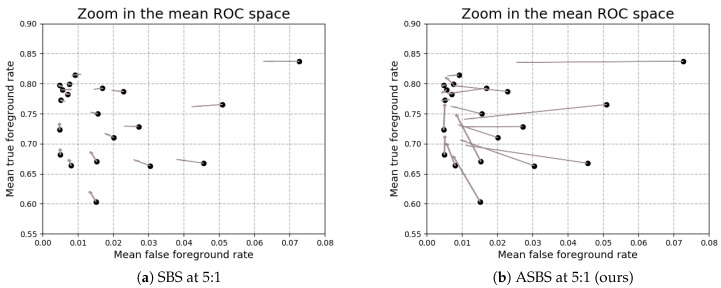
Effects of SBS and ASBS on BGS algorithms in the mean ROC space of CDNet 2014 [12]. Each point represents the performance of a BGS algorithm and the end of the associated arrow indicates the performance after application of the methods for a temporal sub-sampling factor of 5:1. We observe that SBS improves the performances, but only marginally, whereas ASBS moves the performances much closer to the oracle (upper left corner).

In Figure 5, we also compare the effects of SBS with copied $B_{t}$ in $D_{t}$ for frames with missing semantics, and ASBS for different BGS algorithms by looking at their performances in the mean ROC space of CDNet 2014 (ROC space where the false and true foreground rates are computed according to the rules of Reference [12]). The points represent the performances of different BGS algorithms whose segmentation maps can be downloaded on the dataset website. The arrows represent the effects of SBS and ASBS for a temporal sub-sampling factor of 5:1. This choice of frame rate is motivated by the fact that it is the frame rate at which PSPNet can produce the segmentation maps on a GeForce GTX Titan X GPU. We observe that SBS improves the performances, but only marginally, whereas ASBS moves the performances much closer to the oracle (upper left corner).

**Figure 6:**
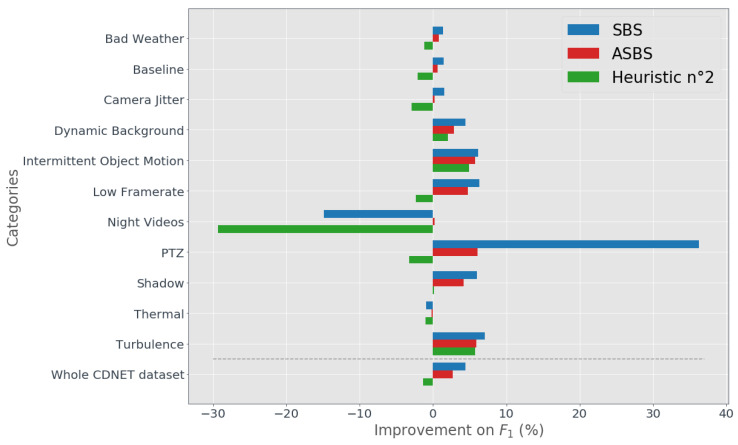
Per-category analysis. We display the relative improvements of the $F_{1}$ score of SBS, ASBS, and the second heuristic compared with the original algorithms, by considering only the frames without semantics (at a 5:1 semantic frame rate).

To better appreciate the positive impact of our strategy for replacing semantics, we also provide a comparative analysis of the $F_{1}$ score by only considering the frames without semantics. We evaluate the relative improvement of the $F_{1}$ score of ASBS, SBS and the second heuristic (SBS with copies of $S_{t}$) compared to the original BGS algorithm (which is equivalent to the first heuristic, SBS with copies of $B_{t}$). In Figure 6, we present our analysis on a per-category basis, in the same fashion as in Reference [30]. As shown, the performances of ASBS are close to the ones of SBS for almost all categories, indicating that our substitute for semantics is adequate. We can also observe that the second heuristic does not perform well, and often degrades the results compared the original BGS algorithm. In this Figure, SBS appears to fail for two categories: “night videos“ and “thermal“. This results from the ineffectiveness of PSPNet to process videos of these categories, as this network is not trained with such image types. Interestingly, ASBS is less impacted than SBS because it refrains from copying some wrong decisions enforced by semantics.

**Figure 7:**
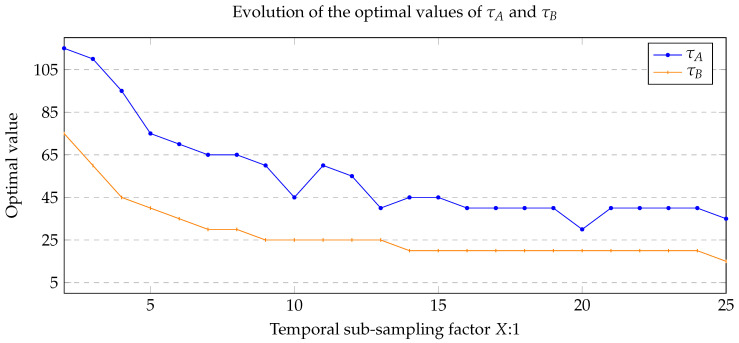
Evolution of the optimal thresholds $\tau_{A}$ and $\tau_{B}$ of the ASBS method when the semantic frame rate is reduced. Note that the Manhattan distance associated to these thresholds is computed on 8-bit color values. The results are shown here for the PAWCS algorithm, and follow the same trend for the IUTIS-5, SuBSENSE, and WeSamBe BGS algorithms.

Finally, in Figure 7, we provide the evolution of the optimal parameters $\tau_{A}$ and $\tau_{B}$ with the temporal sub-sampling factor (in the case of PAWCS). The optimal value decreases with the sub-sampling factor, implying that the matching condition on colors become tighter or, in other words, that ruleA and ruleB should be activated less frequently for lower semantic frame rates, as a consequence of the presence of more outdated colors in the color map for further images.

### 4.3. A Feedback Mechanism for SBS and ASBS

The methods SBS and ASBS are designed to be combined to a BGS algorithm to improve the quality of the final segmentation, but they do not affect the decisions taken by the BGS algorithm itself. In this section, we explore possibilities to embed semantics inside the BGS algorithm itself, which would remain blind to semantics otherwise. Obviously, this requires to craft modifications specific to a particular algorithm or family of algorithms, which can be effortful as explained hereinafter.

The backbone of many BGS algorithms is composed of three main parts. First, an internal model of the background is kept in memory, for instance in the form of color samples or other types of features. Second, the input frame is compared to this model via a distance function to classify pixels as background or foreground. Third, the background model is updated to account for changes in the background over time.

**Figure 8:**
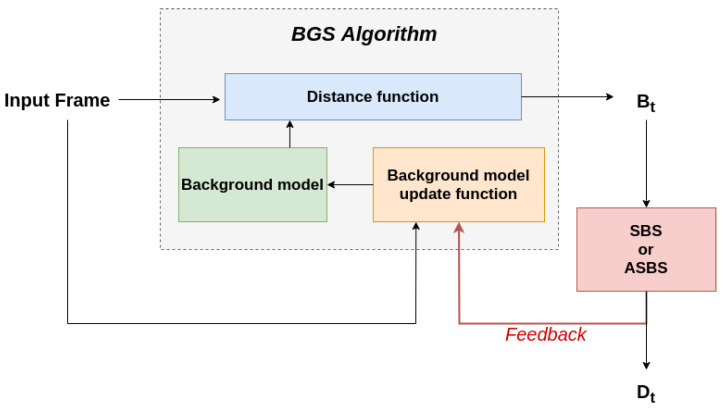
Our feedback mechanism, which impacts the decisions of any BGS algorithm whose model update is conservative, consists to replace the BG/FG segmentation of the BGS algorithm by the final segmentation map improved by semantics (either by SBS or ASBS) to update the internal background model.

A first possibility to embed semantics inside the BGS algorithm is to include semantics directly in a joint background model integrating color and semantic features. This requires to formulate the relationships that could exist between them and to design a distance function accounting for these relationships, which is not trivial. Therefore, we propose a second way of doing so by incorporating semantics during the update, which is straightforward for algorithms whose model updating policy is conservative (as introduced in Reference [5]). For those algorithms, the background model in pixel (x,y) may be updated if $B_{t}\left(x,y\right)=\mathrm{BG}$, but it is always left unchanged if $B_{t}\left(x,y\right)=\mathrm{FG}$, which prevents the background model from being corrupted with foreground features. In other words, the segmentation map $B_{t}$ serves as an updating mask. As $D_{t}$ produced by SBS or ASBS is an improved version of $B_{t}$, we can advantageously use $D_{t}$ instead of $B_{t}$ to update the background model, as illustrated in Figure 8. This introduces a semantic feedback which improves the internal background model and, consequently, the next segmentation map $B_{t+1}$, whether or not semantics is computed.

To appreciate the benefit of a semantic feedback, we performed experiments for two well-known conservative BGS algorithms, ViBe and SuBSENSE, using the code made available by the authors (see Reference [37] for ViBe and Reference [38] for SuBSENSE). Let us note that the performances for SuBSENSE are slightly lower than the ones reported in Figure 4 as there are small discrepancies between the performance reported on the CDNet web site and the ones obtained with the available source code.

**Figure 9:**
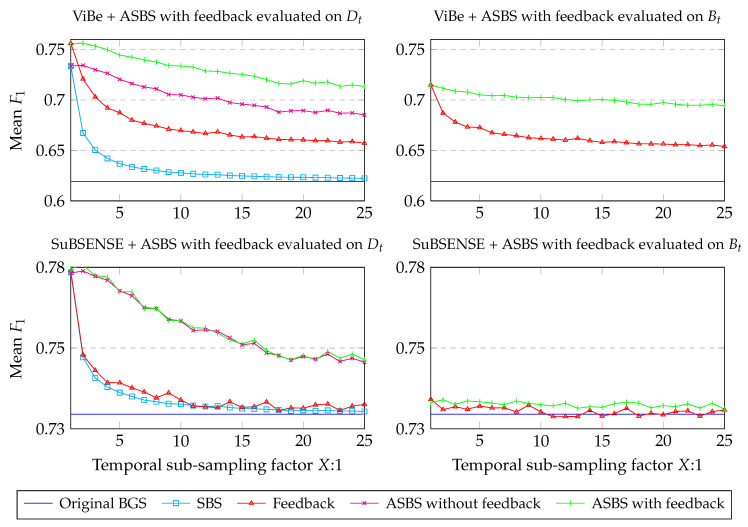
Comparison of the performances, computed with the mean $F_{1}$ score on the CDNet 2014, of SBS and ASBS when there is a feedback that uses $D_{t}$ to update the model of the BGS algorithm. The results are given with respect to a decreasing semantic frame rate. It can be seen that SBS and ASBS always improve the results of the original BGS algorithm and that a feedback is beneficial. Graphs in the right column show that the intrinsic quality of the BGS algorithms is improved, as their output $B_{t}$, prior to any combination with semantics, produces higher mean $F_{1}$ scores.

Figure 9 (left column) reports the results of ASBS with the feedback mechanism on ViBe and SuBSENSE, and compares them to the original algorithm and the SBS method. Two main observations can be made. First, as for the results of the previous section, SBS and ASBS both improve the performances even when the semantic frame rate is low. Also, ASBS always performs better. Second, including the feedback always improves the performances for both SBS and ASBS, and for both BGS algorithms. In the case of ViBe, the performance is much better when the feedback is included. For SuBSENSE, the performance is also improved, but only marginally. This might be due to the fact that ViBe has a very straightforward way of computing the update of the background model while SuBSENSE uses varying internal parameters and heuristics, calculated adaptively. It is thus more difficult to interpret the impact of a better updating map on SuBSENSE than it is on ViBe.

We also investigated to what extend the feedback provides better updating maps to the BGS algorithm. For conservative algorithms, this means that, internally, the background model is built with better features. This measure can be evaluated using the output of the classification map, $B_{t}$.

For that purpose, we compared the original BGS algorithm and the direct output, that is $B_{t}$ in Figure 8, of the feedback method when the updating map is replaced by $D_{t}$ obtained by either SBS or ASBS. As can be seen in Figure 9 (right column), using the semantic feedback always improves the BGS algorithm whether the updating map is obtained from SBS or ASBS. This means that the internal background model of the BGS algorithm is always enhanced and that, consequently, a feedback helps the BGS algorithm to take better decisions.

**Figure 10:**
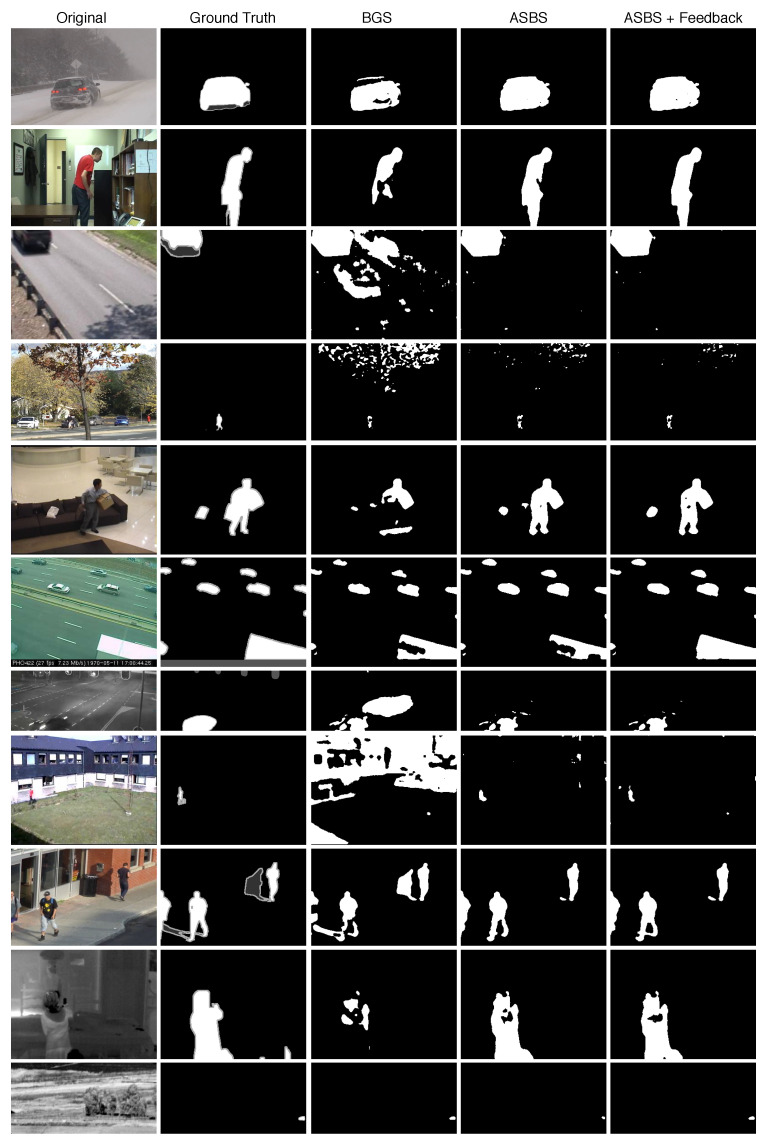
Illustration of the results of ASBS using ViBe as BGS algorithm. From left to right, we provide the original color image, the ground truth, the BGS as provided by the original ViBe algorithm, using our ASBS method without any feedback, and using ASBS and a feedback. Each line corresponds to a representative frame of a video in each category of CDNet2014.

Finally, let us note that ViBe, which is a real-time BGS algorithm, combined with semantics provided at a real-time rate (about 1 out of 5 frames) and with the feedback from ASBS has a mean $F_{1}$ performance of 0.746, which is the same performance as the original SuBSENSE algorithm that is not real time [33]. This performance corresponds to the performance of RT-SBS presented in [31]. It can be seen that our method can thus help real-time algorithms to reach performances of the top unsupervised BGS algorithms while meeting the real-time constraint, which is a huge advantage in practice. We illustrate our two novel methods, ASBS and the feedback, in Figure 10 on one video of each category of the CDNet2014 dataset using ViBe as BGS algorithm.

One last possible refinement would consist to adapt the updating rate of the background model according to a rule map similar to that of ASBS. More specifically, if $B_{t}\left(x,y\right)=\mathrm{FG}$ and $D_{t}\left(x,y\right)=\mathrm{BG}$, we could assume that the internal background model in pixel (x,y) is inadequate and, consequently, we could increase the updating rate in that pixel. Tests performed on ViBe showed that the performances are improved with this strategy. However, this updating rate adaptation has to be tailored for each BGS algorithm specifically; therefore, we did not consider this final refinement in our experiments. We only evaluated the impact of the feedback mechanism on BGS algorithms with a conservative updating policy, and avoided any particular refinement that would have biased the evaluation.

### 4.4. Time Analysis of ASBS 

In this section, we show the timing diagram of ASBS and provide typical values for the different computation durations.

**Figure 11:**
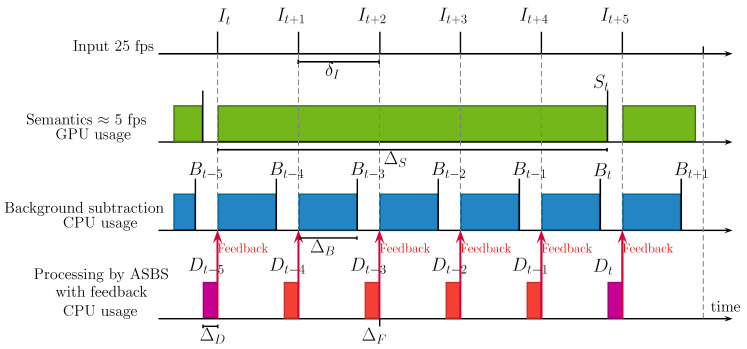
Timing diagram of ASBS with a feedback mechanism in the case of a real-time BGS algorithm ($\Delta_{B}<\delta_{I}$) satisfying the condition $\Delta_{B}+\Delta_{D}<\delta_{I}$ and the computation of semantics being not real-time ($\Delta_{S}>\delta_{I}$). Note that the feedback time $\Delta_{F}$ is negligible.

The timing diagram of ASBS with feedback is presented in Figure 11. The inclusion of a feedback has two effects. First, we need to include the feedback time $\Delta_{F}$ in the time needed for the background subtraction algorithm $\Delta_{B}$. In our case, as we only substitute the updating map by $D_{t}$, it can be implemented as a simple pointer replacement and therefore $\Delta_{F}$ is negligible (in the following, we take $\Delta_{F}≃0\;\mathrm{ms}$). Second, we have to wait for ASBS (or SBS) to finish before starting the background subtraction of the next frame.

Concerning the computation time of BGS algorithms, Roy et al. [33] have provided a reliable estimate of the processing speed of leading unsupervised background subtraction algorithms. They show that the best performing ones are not real time. Only a handful of algorithms are actually real time, such as ViBe that can operate at about 200fps on CDNet 2014 dataset, that is $\Delta_{B}=5\;\mathrm{ms}$. With PSPNet, the semantic frame rate is of about 5 to 7fps for a NVIDIA GeForce GTX Titan X GPU, which corresponds to $\Delta_{S}≃200\;\mathrm{ms}$. It means that for 25fps videos, we have access to semantics about once every 4 to 5 frames. In addition, Table 3 reports our observation about the mean execution time per frame of $\Delta_{D}$ for SBS and ASBS. These last tests were performed on a single thread running on a single processor Intel(R) Xeon(X) E5-2698 v4 2.20GHz.

Thus, in the case of ViBe, we start from a frame rate of about 200fps in its original version to reach about 160fps when using ASBS, which is still real time. This is important because, as shown in Section 4.3, the performances of ViBe with ASBS at a semantic frame rate of 1 out of 5 frames and feedback is the same as SuBSENSE that, alone, runs at a frame rate lower than 25fps [33]. Hence, thanks to ASBS, we can replace BGS algorithms that work well but are too complex to run in real time and are often difficult to interpret by a combination of a much simpler BGS algorithm and a processing based on semantics, regardless of the frame rate of the last. Furthermore, ASBS is much easier to optimize as the parameters that we introduce are few in number and easy to interpret. In addition, we could also fine-tune the semantics, by selecting a dedicated set of objects to be considered, for a scene-specific setup. It is our belief that there are still some margins for further improvements.

## 5. Conclusions

In this paper, we presented a novel method, named ASBS, based on semantics for improving the quality of segmentation masks produced by background subtraction algorithms when semantics is not computed for all video frames. ASBS, which is derived from the semantic background subtraction method, is applicable to any off-the-shelf background subtraction algorithm and introduces two new rules in order to repeat semantic decisions, even when semantics and the background are computed asynchronously. We also presented a feedback mechanism to update the background model with better samples and thus take better decisions. We showed that ASBS improves the quality of the segmentation masks compared to the original semantic background subtraction method applied only to frames with semantics. Furthermore, ASBS is straightforward to implement and cheap in terms of computation time and memory consumption. We also showed that applying ASBS with the feedback mechanism allows to elevate an unsupervised real-time background subtraction algorithm to the performance of non real-time state-of-the-art algorithms.

A more general conclusion is that, when semantics is missing for some frames but needed to perform a task (in our case, the task of background subtraction), our method provides a convenient and effective mechanism to interpolate the missing semantics. The mechanism of ASBS might thus enable real-time computer vision tasks requiring semantic information.

Implementations of ASBS in the Python language for CPU and GPU are available at the following address https://github.com/cioppaanthony/rt-sbs.

## Figures and Tables

**Table 1:** Comparison of the best mean $F_{1}$ score achieved for two semantic networks used in combination with SBS on the CDNet 2014 dataset. These performances are obtained considering the SBS method, where the output of the BGS algorithm is replaced by the ground-truth masks. This indicates how the semantic information used in SBS would deteriorate a perfect BGS algorithm.

Networks	SBS with PSPNet [25]	SBS with MaskRCNN [26]
Best mean $F_{1}$	0.953	0.674

**Table 2:** Decision table as implemented by SBS. Rows corresponding to “don’t-care” values (X) cannot be encountered, assuming that $\tau_{\mathrm{BG}}<\tau_{\mathrm{FG}}$.

$B_{t}\left(x,y\right)$	$S_{t}^{\mathrm{BG}}\left(x,y\right)\leq \tau_{\mathrm{BG}}$	$S_{t}^{\mathrm{FG}}\left(x,y\right)\geq \tau_{\mathrm{FG}}$	$D_{t}\left(x,y\right)$
BG	false	false	BG
BG	false	true	FG
BG	true	false	BG
BG	true	true	X
FG	false	false	FG
FG	false	true	FG
FG	true	false	BG
FG	true	true	X

**Table 3:** Mean computation time $\Delta_{D}$ (ms/frame) of SBS and ASBS.

$\Delta_{D}\left(\mathrm{SBS}\right)$	1.56
$\Delta_{D}\left(\mathrm{ASBS}:\;\mathrm{frames}\;\mathrm{with}\;\mathrm{semantics}\right)$	2.12
$\Delta_{D}\left(\mathrm{ASBS}:\;\mathrm{frames}\;\mathrm{without}\;\mathrm{semantics}\right)$	0.8
